# Assessing the Quality of Milk Using a Multicomponent Analytical Platform MicroNIR/Chemometric

**DOI:** 10.3389/fchem.2020.614718

**Published:** 2020-12-01

**Authors:** Roberta Risoluti, Giuseppina Gullifa, Stefano Materazi

**Affiliations:** Department of Chemistry, Sapienza University of Rome, Rome, Italy

**Keywords:** milk, microNIR, chemometrics, quality control, new methods, multiparametric analysis

## Abstract

In this work, an innovative screening platform based on MicroNIR and chemometrics is proposed for the on-site and contactless monitoring of the quality of milk using simultaneous multicomponent analysis. The novelty of this completely automated tool consists of a miniaturized NIR spectrometer operating in a wireless mode that allows samples to be processed in a rapid and accurate way and to obtain in a single click a comprehensive characterization of the chemical composition of milk. To optimize the platform, milk specimens with different origins and compositions were considered and prediction models were developed by chemometric analysis of the NIR spectra using Partial Least Square regression algorithms. Once calibrated, the platform was used to predict samples acquired in the market and validation was performed by comparing results of the novel platform with those obtained from the chromatographic analysis. Results demonstrated the ability of the platform to differentiate milk as a function of the distribution of fatty acids, providing a rapid and non-destructive method to assess the quality of milk and to avoid food adulteration.

## Introduction

Milk is one of the most investigated food matrices worldwide and the analyses usually aim at estimating its chemical composition, in order to evaluate its quality (Hambraeus, 1984; Cunsolo et al., 2017). In particular, the quality of milk is mainly related to the fatty acid content (Napoli et al., 2007; Zhou et al., 2014), as well as the amount of lactose, proteins, and vitamin D. The increasing demand from consumers for innovative dietary products has globally led to an increasing request of procedures and methods to detect adulteration of food matrices (Cossignani et al., 2011; Materazzi et al., 2012). In particular, adulteration of milk mainly consists of the addition or the illegal use of additives or molecules, including melamine (Balabin and Smirnov, 2011), sugars (Kamboj et al., 2020), urea (Mabood et al., 2019), and formalin (Saha and Thangavel, 2018).

Therefore, a method which could assess the quality of a food product and perform a multicomponent characterization at the first level test represents an important issue when dealing with the monitoring of food quality or human health. For this reason, innovative screening systems, able to rapidly process samples without requiring any pretreatment or clean up, in a non-destructive way, are becoming more and more recommended (Dunn et al., 2011; Risoluti et al., 2016, 2019a,b,c; D'Elia et al., 2018). Reference analytical procedures for milk analyses usually require chromatographic techniques, such as High Performance Liquid Chromatography associated to Mass Spectrometry (HPLC-MS) (Chotyakul et al., 2014; Aiello et al., 2015; Rocchetti et al., 2020), Gas Chromatography coupled to Mass Spectrometry (GC-MS) (Marchetti et al., 2002; Materazzi and Risoluti, 2014; Teng et al., 2017), and Nuclear Magnetic Resonance (NMR) (Garcia et al., 2012; Crea et al., 2014; Santos et al., 2016; Aiello et al., 2018).

Spectroscopic techniques are widely recognized as solvent-free, fast, and easy-to-use tools to perform the chemical investigation of different matrices without destroying samples (Oliveri et al., 2011; Materazzi et al., 2017a,b; Véstia et al., 2019). In particular, NIR spectroscopy associated with chemometric analysis proved its high potential in multicomponent analyses, at low costs and without requiring the supervision of specialized personnel (Kurdziel et al., 2003; Migliorati et al., 2013; Kordi et al., 2017; Materazzi et al., 2017c; Mees et al., 2018). NIR spectroscopy has been largely proposed for the investigation of milk with the aim of providing innovative and rapid methods for the detection of lactose, proteins, carotenoids, and fatty acid contents (Jankovskà and Šustovà, 2003; Numthuam et al., 2017; Risoluti et al., 2017; Wang et al., 2019; Soulat et al., 2020). In addition, NIR spectroscopy allows users moving out of the laboratory and performing prediction of analytes in complex matrices (Navarra et al., 2003; Bretti et al., 2013; Paiva et al., 2015; Basri et al., 2017; da Silva et al., 2017; Modrono et al., 2017; Risoluti and Materazzi, 2018; Risoluti et al., 2018a,b) by means of portable instruments that directly analyze milk and provide the results (De Angelis Curtis et al., 2008; Bian et al., 2017; Ferreira de Lima et al., 2018). Despite these instruments permitting the transfer of validated methods directly on site, they do not provide a tool enabling consumers to rapidly check the product by itself for application to real situations.

Based on these considerations, this work proposes a novel method based on a miniaturized spectrometer, the MicroNIR OnSite, for the multicomponent analysis of milk for food quality control. This platform uses chemometric tools to develop models of prediction that, once validated, provide the fast and accurate characterization of milk specimens in a “click,” using a contactless and wireless miniaturized instrument that can be installed on a consumer's smartphone.

## Materials and Methods

### Experimental

#### Analytical Workflow

Milk specimens were provided by different producers in Italy and included cow, goat, and donkey milk, as well as rice milk. In addition, samples were selected considering their different amounts of fats and treatments and considering whole, skimmed, and low-fat milk and UHT milk. For each sample, about 1 ml of milk was directly analyzed by the MicroNIR equipped with a special accessory for liquids; no sample pre-treatment was necessary. A detailed list of the investigated samples is reported in Table 1.

**Table 1:** List of the investigated milk specimens.

**Samples**	**Number of spectra**	**Fats (g/100 mL)**
**Whole Milk**		
Goat Milk—Fresh Whole Milk—Amalattea	100	3.9
Goat Milk—Latte UHT Intero—Amalattea	150	3.9
Cow Milk—Fresh Whole Milk—Centrale del Latte	100	3.6
Cow Milk—Fresh Organic Whole Milk—Centrale del Latte	100	3.6
**Semi Skimmed Milk**		
Goat Milk—Semi Skimmed UHT Milk—Amalattea	150	1.6
Cow Milk—Fresh Semi Skimmed Milk—Centrale del Latte	150	1.6
Cow Milk—Semi Skimmed Organic UHT Milk—Granarolo	150	1.6
Cow Milk—Semi Skimmed UHT Milk—Zymil Parmalat	150	1
Cow Milk—Semi Skimmed Organic UHT Milk—Zymil Parmalat	150	1
Cow Milk—Microfiltered Semi Skimmed UHT Milk—Selex	150	1
Cow Milk—Semi Skimmed UHT Milk—Accadì	50	1
**Skimmed Milk**		
Cow Milk—Fresh Skimmed Milk—Centrale del Latte	50	<0.5
Cow Milk—Skimmed UHT Milk—Parmalat	150	<0.5
Cow Milk—Skimmed UHT Milk—Centrale del Latte	100	0.1
Cow Milk—Skimmed UHT Milk—Zymil Parmalat	150	0.1
Cow Milk—Skimmed UHT Milk—Accadì	100	0.1
**Rice Milk**		
UHT Rice Milk—Vital Nature	100	1
**Donkey Milk**		
UHT Donkey Milk	50	

Chemometric analysis was performed by Principal Component Analysis in order to evaluate correlations among measurements and to provide a rapid tool able to identify the adulteration of milk as a function of the fats contents.

Calibration and validation of the platform was obtained by dividing the data set of measurements in the training set and evaluation set, while the prediction of real samples was achieved by processing 17 additional samples not previously included in the dataset and thus processed as an independent batch. This step is strictly required, in order to guarantee the results are not bath-dependent and to ensure reproducibility and effectiveness of the platform.

#### MicroNIR On-Site Spectrometer

The MicroNIR On-Site is a portable spectrometer device operating in the NIR region of the spectrum (900–1,700 nm) and distributed by Viavi Solutions (JDSU Corporation, Milpitas, USA). It is specifically the latest version of the ultracompact MicroNIR from Viavi and represents the real update in the field of the miniaturized device, moving out of the laboratory. In fact, it is provided by two different pieces of software (JDSU Corporation, Milpitas, USA): the first is the MicroNIR Pro software that allows trained users to collect samples and develop a model of prediction; the second is the MicroNIR OnSite-W system for real-time prediction of samples and it is suitable even for untrained users.

Calibration of the instrument was obtained prior to the acquisition of the sample, by means of a special accessory that permitted the registration of a dark reference (total absorbance) and a white reference (total reflectance) using Spectralon. The instrumental settings included a nominal spectral resolution of the acquisitions at 6.25 nm and an integration time of 10 ms, for a total measurement time of 2.5 s per sample. Chemometric analysis was performed by V-JDSU Unscrambler Lite (Camo software AS, Oslo, Norway). Ten spectra for each sample were collected in order to ensure heterogeneity of the measurement and the mean was considered for the chemometric analysis. The investigation of samples' correlation was first performed by Principal Component Analysis and the models of prediction were developed by the mean of Partial Least Square regression algorithms.

## Results and Discussion

The feasibility of innovative techniques to address specific issues strictly relies on the standardization of the method on reference samples as representative as possible of those to be processed. To this aim, a reference dataset of samples was considered by processing a number of different kinds of milk, such as cow, goat, and donkey, with different fatty acid contents. In addition, milk specimens after UHT treatments were also included, in order to provide a comprehensive method able to be used for a variety of products. With the aim of avoiding a batch dependent response of the analytical platform, different batches of the same milk and different providers were considered.

**Figure 1:**
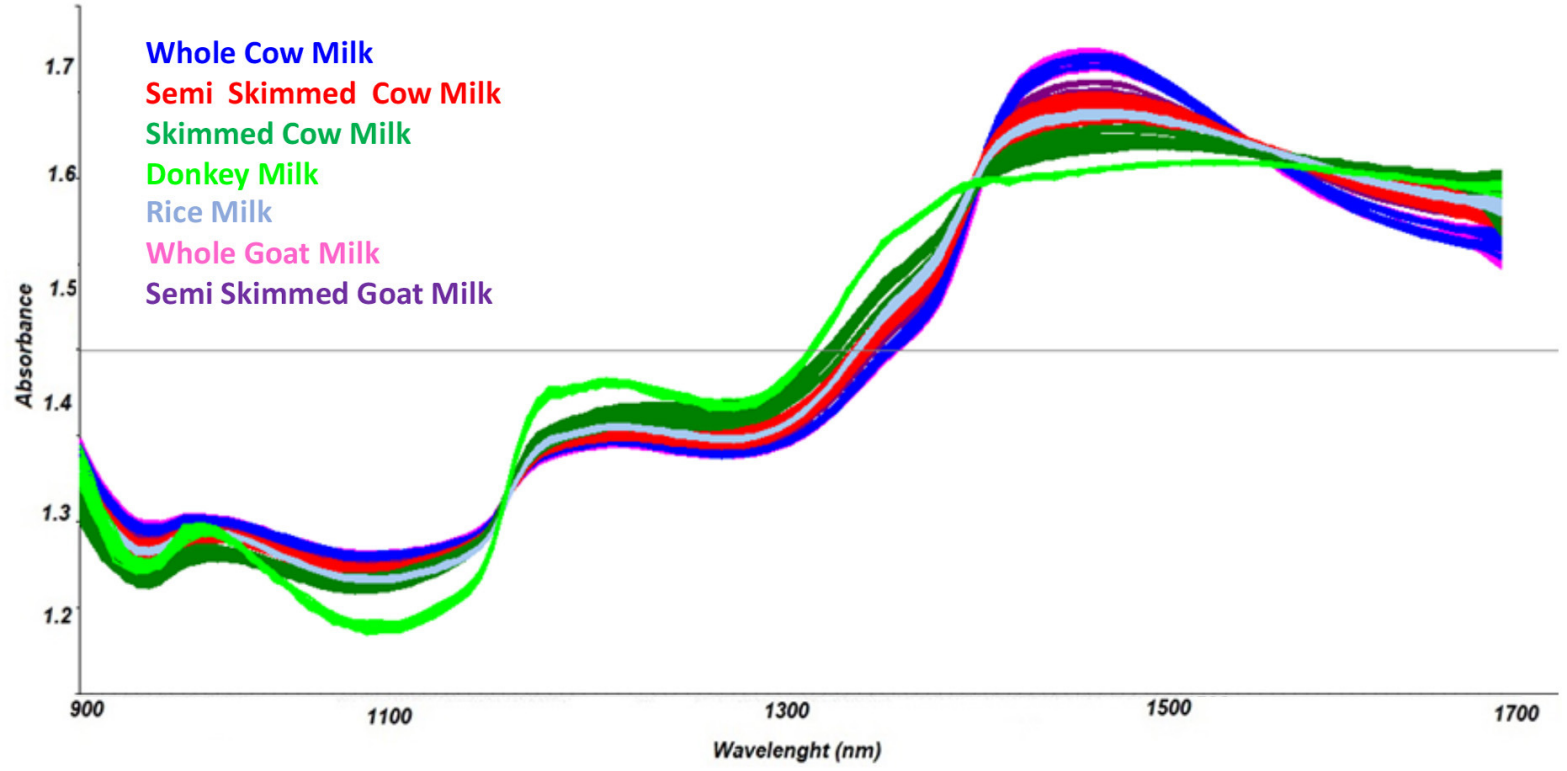
Collected spectra of milk specimens.

Spectra in the NIR region were recorded by the MicroNIR OnSite device, as reported in Figure 1, and chemometric pre-treatments were investigated in order to separate samples according to the different types of milk.

Mathematical transformations usually recommended for spectroscopic data (Barnes et al., 1989) were evaluated; in particular, scatter-correction methods were applied, such as Standard Normal Variate transform (SNV) (Geladi et al., 1985), Multiplicative Scatter Correction (MSC), Mean Centering (MC) (Wold and Sjöström, 1977), and normalization (Savitzky and Golay, 1964). In addition, spectral derivation techniques, including Savitzky-Golay (SG) polynomial derivative filters (Rinnan et al., 2009) were considered. Among the investigated spectra pre-treatments, combination of the baseline correction, first derivative transform, and Multiplicative Scatter Correction (MSC) were used to highlight differences among spectra and thus to separate samples according to the different chemical compositions.

**Figure 2:**
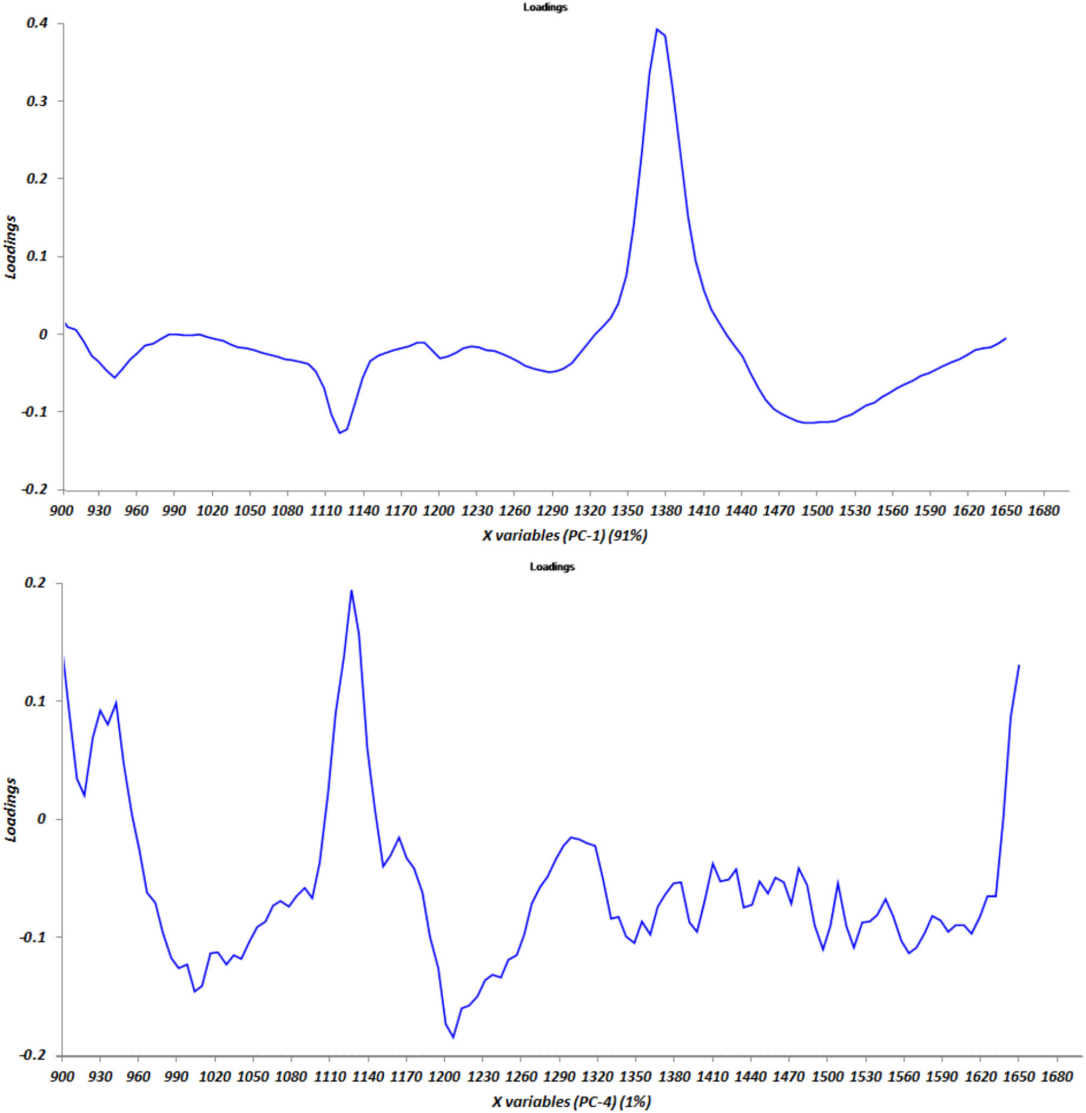
Graph of the loading factors vs. principal components PC 1 and PC4.

Results of the NIR spectra interpretation from Sýasic and Ozaki (2001) were confirmed, as shown in Figure 2, where the graph of the loadings vs. components PC1 and PC4 is reported.

**Figure 3:**
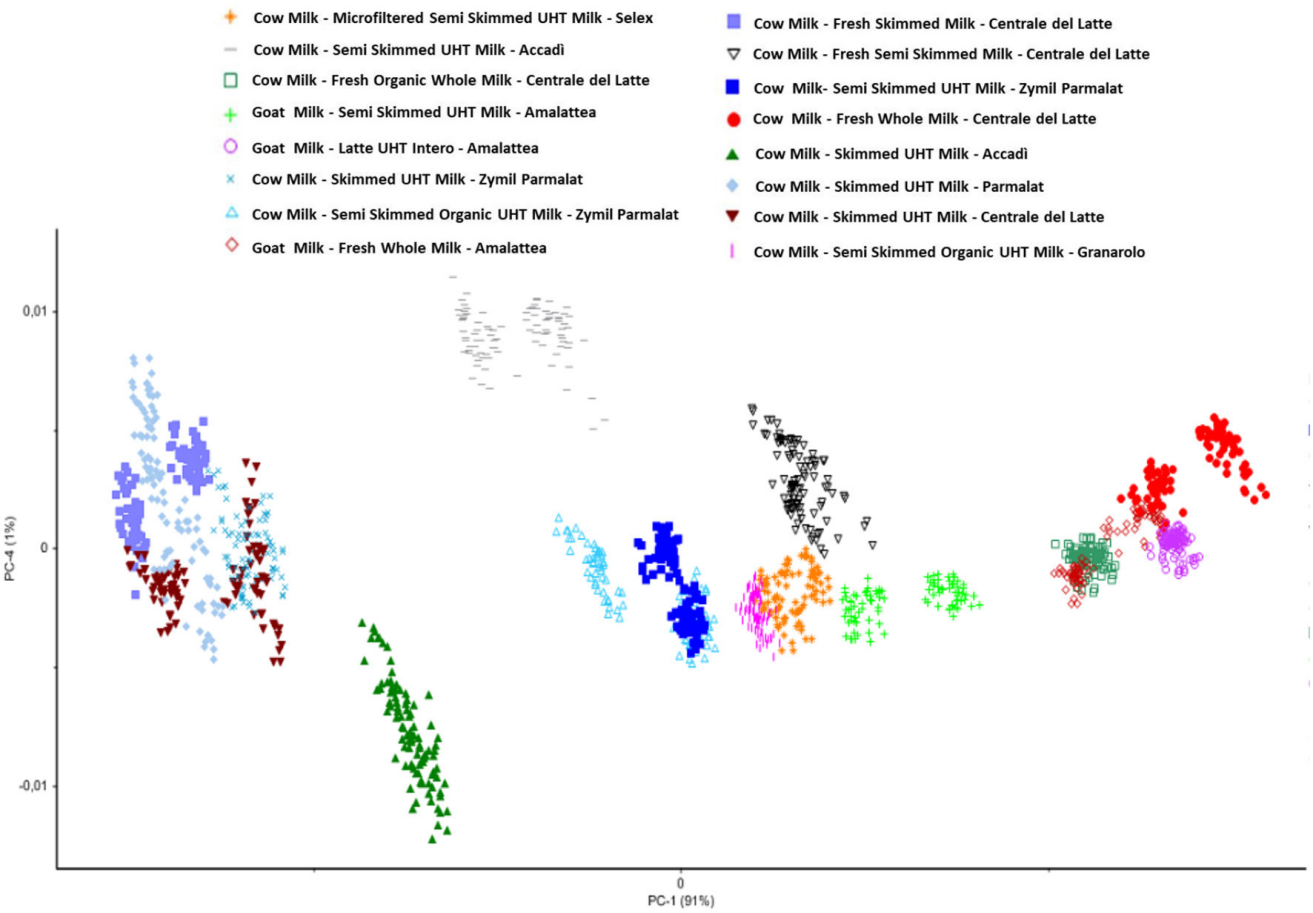
Principal component analysis performed on all the collected samples.

**Figure 4:**
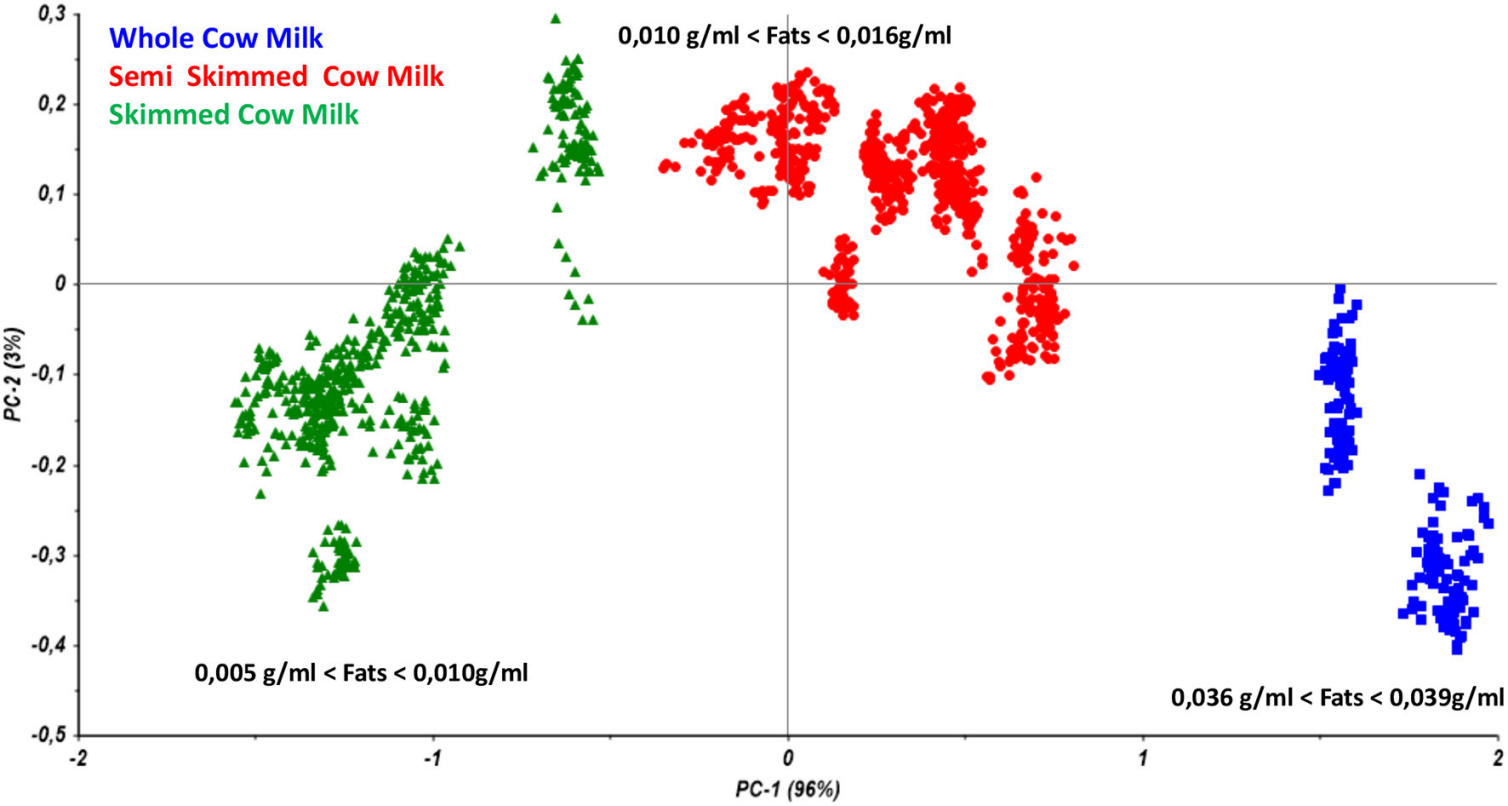
Principal component analysis performed on all the collected samples from cow milk with different fats content.

Therefore, the preliminary Principal Component Analysis performed on the entire dataset of measurements shows a good accordance among samples belonging to the same class (Figure 3) and shows a distribution of the samples as a function of the increasing fatty acid content (Figure 4).

This behavior led us to develop a quantitation model by considering a Partial Least Square Regression algorithm in order to develop a model of predicting milk that permits the rapidly evaluation of its origin and its quality. As required for the validation of analytical methods, all the collected spectra were divided into a training set (about 75% of samples) and evaluation set (about 25% of samples), and a number of parameters were assessed in order to calculate the model's performances.

Among these, the Root Mean Squared Errors (RMSEs) and the correlation coefficient (R^2^) were estimated in calibration and cross-validation by using seven latent variables, while precision and sensitivity were calculated to provide fast and accurate outcomes when dealing with real samples analysis.

**Figure 5:**
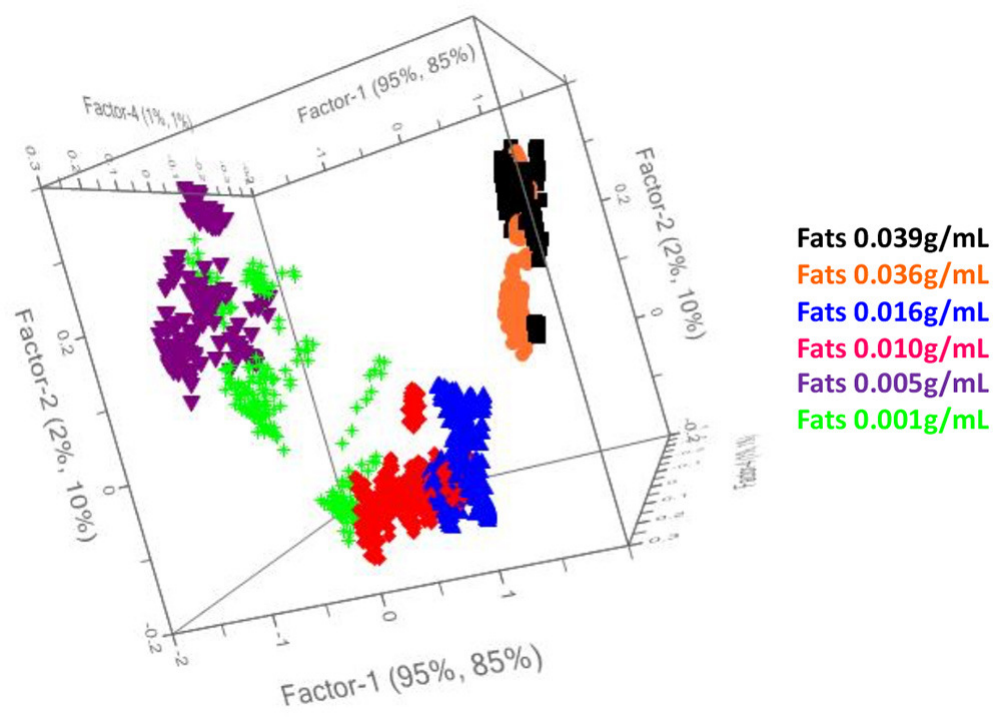
PLS model for fat quantitation in milk specimens.

As reported in Figure 5, the model allows users to simultaneously differentiate whole, semi-skimmed, and skimmed milk and to identify the different origins.

As a consequence, the model may recognize the milk quality as a function of the belonging cluster of the spectra and preliminarily addresses the subsequent analyses.

The prediction ability of the model was evaluated by estimating the figures of merit and satisfactory outcomes may be observed; in fact, the correlation values were never lower than 0.99 in calibration or in cross-validation.

In addition, accuracy, precision, the slope, and the Root Mean Square Error (RMSE) were calculated considering seven latent variables. Results are summarized in Table 2.

**Table 2:** PLS model for milk: estimation of the figures of merit in calibration and cross-validation.

	**Calibration**	**Cross-validation**
Slope	0.97	0.97
*R*^2^	0.099	0.98
RMSE (g/mL)	0.002	0.002
Accuracy (%)	98.4	97.1
Precision (%)	75.3	74.6
LVs	7	7

Satisfactory performances of the model may be observed, leading to errors in cross-validation no higher than 0.002 g/mL and accuracy values no lower than 97.1%. Precision of the method was also calculated and suitable outcomes were obtained, resulting in precision values about 74.6%.

### Feasibility of the Platform

Prediction of the real samples was performed by processing 17 milk specimens commercially available in the Italian markets, in order to evaluate the platform performances. Samples were analyzed by the MicroNIR On-Site and spectra were processed by the optimized chemometric model. Good accordance among measurements was observed from MicroNIR outcomes, as reported in Table 3. The graph of the measured vs. predicted samples provided for a correlation coefficient of about 0.996, as all the samples were correctly predicted by the model, confirming the promising application of the platform.

**Table 3:** Results obtained from the novel platform for independent real samples.

**Prediction results**	**Fats (g/mL)**	**Predicted**	**DS**
Goat milk–Fresh whole milk–Amalattea	0.039	0.0422	0.0002
Goat milk–Latte UHT intero–Amalattea	0.039	0.0372	0.0005
Cow milk–Fresh whole milk–Centrale del Latte	0.036	0.0337	0.0003
Cow milk–Fresh organic whole milk–Centrale del Latte	0.036	0.0331	0.0004
Goat milk–Semi skimmed UHT milk–Amalattea	0.016	0.0178	0.0004
Cow milk–Fresh semi skimmed milk–Centrale del Latte	0.016	0.0130	0.0005
Cow milk–Semi skimmed organic UHT milk–Granarolo	0.016	0.0139	0.0004
Cow milk–Semi skimmed UHT milk–Zymil Parmalat	0.01	0.0083	0.0003
Cow milk–Semi skimmed organic UHT milk–Zymil Parmalat	0.01	0.0089	0.0004
Cow milk–Microfiltered Semi skimmed UHT milk–Selex	0.01	0.0140	0.0005
Cow milk–Semi skimmed UHT milk–Accadì	0.01	0.0084	0.0004
UHT Rice milk–Vital Nature	0.01	0.0108	0.0004
Cow milk–Fresh Skimmed milk–Centrale del Latte	0.005	0.0067	0.0003
Cow milk–Skimmed UHT milk–Parmalat	0.005	0.0042	0.0006
Cow milk–Skimmed UHT milk–Centrale del Latte	0.001	0.0030	0.0003
Cow milk–Skimmed UHT milk–Zymil Parmalat	0.001	0.0011	0.0004
Cow milk–Skimmed UHT milk–Accadì	0.001	0.0032	0.0004

## Conclusions

In this work, a novel analytical platform based on NIR spectroscopy and chemometrics is proposed for the monitoring of milk quality. The novelty of the platform is strictly related to the innovative MicroNIR On-Site device which can be used to collect samples and to perform the prediction in few seconds, even by untrained personnel with an automated platform available on a smartphone. Reliability of this novel test was assessed by processing independent real samples, confirming the feasibility of this novel platform. In addition, the model was validated by estimating the characteristic figures of merit, such as the accuracy, slope, precision, and the RMSE, demonstrating its suitability for application as a screening test for consumers for food monitoring.

## Data Availability Statement

The raw data supporting the conclusions of this article will be made available by the authors, without undue reservation.

## Author Contributions

SM and RR conceived the study and interpreted the data by chemometrics and GG performed the analyses. The manuscript was written through contributions of all authors. All authors have given approval to the final version of the manuscript.

## Conflict of Interest

The authors declare that the research was conducted in the absence of any commercial or financial relationships that could be construed as a potential conflict of interest.
